# Development of a deep learning method for improving diagnostic accuracy for uterine sarcoma cases

**DOI:** 10.1038/s41598-022-23064-5

**Published:** 2022-11-16

**Authors:** Yusuke Toyohara, Kenbun Sone, Katsuhiko Noda, Kaname Yoshida, Ryo Kurokawa, Tomoya Tanishima, Shimpei Kato, Shohei Inui, Yudai Nakai, Masanori Ishida, Wataru Gonoi, Saki Tanimoto, Yu Takahashi, Futaba Inoue, Asako Kukita, Yoshiko Kawata, Ayumi Taguchi, Akiko Furusawa, Yuichiro Miyamoto, Takehiro Tsukazaki, Michihiro Tanikawa, Takayuki Iriyama, Mayuyo Mori-Uchino, Tetsushi Tsuruga, Katsutoshi Oda, Toshiharu Yasugi, Kimihiro Takechi, Osamu Abe, Yutaka Osuga

**Affiliations:** 1 Department of Obstetrics and Gynecology, Graduate School of Medicine, Faculty of Medicine, The University of Tokyo, 7-3-1 Hongo Bunkyo-Ku, Tokyo, 113-8655 Japan; 2 SIOS Technology, Inc., Tokyo, Japan; 3 Department of Radiology, Graduate School of Medicine, The University of Tokyo, Tokyo, Japan; 4 Department of Obstetrics and Gynecology, Tokyo Metropolitan Cancer and Infectious Diseases Center Komagome Hospital, Tokyo, Japan; 5 Department of Obstetrics and Gynecology, Showa General Hospital, Tokyo, Japan; 6 Division of Integrative Genomics, Graduate School of Medicine, The University of Tokyo, Tokyo, Japan

**Keywords:** Medical research, Oncology

## Abstract

Uterine sarcomas have very poor prognoses and are sometimes difficult to distinguish from uterine leiomyomas on preoperative examinations. Herein, we investigated whether deep neural network (DNN) models can improve the accuracy of preoperative MRI-based diagnosis in patients with uterine sarcomas. Fifteen sequences of MRI for patients (uterine sarcoma group: n = 63; uterine leiomyoma: n = 200) were used to train the models. Six radiologists (three specialists, three practitioners) interpreted the same images for validation. The most important individual sequences for diagnosis were axial T2-weighted imaging (T2WI), sagittal T2WI, and diffusion-weighted imaging. These sequences also represented the most accurate combination (accuracy: 91.3%), achieving diagnostic ability comparable to that of specialists (accuracy: 88.3%) and superior to that of practitioners (accuracy: 80.1%). Moreover, radiologists’ diagnostic accuracy improved when provided with DNN results (specialists: 89.6%; practitioners: 92.3%). Our DNN models are valuable to improve diagnostic accuracy, especially in filling the gap of clinical skills between interpreters. This method can be a universal model for the use of deep learning in the diagnostic imaging of rare tumors.

## Introduction

Uterine sarcomas are rare, occurring in approximately 5 in 10,000 women^1^. Although various treatment methods have been proposed—including surgery, chemotherapy, radiotherapy, hormone therapy, and immunotherapy––prognosis among patients with uterine sarcoma remains very poor. Indeed, despite some variation based on histopathological type, the 5-year overall survival rate does not typically reach 50%, especially among patients in the advanced stages^2–4^.

The term “uterine sarcoma” is usually exclusive of carcinosarcoma, which is epithelial in origin and is associated with a relatively better response to treatment and more favorable prognosis when compared with other types of sarcomas (in this report, "uterine sarcoma" refers to sarcoma types other than carcinosarcoma)^5^. Leiomyosarcoma (LMS) represents the major histopathological type of uterine sarcoma, accounting for approximately 60% of cases, followed by endometrial stromal sarcoma (ESS) and adenosarcoma^5^. In addition, smooth muscle tumors that cannot be diagnosed as benign or malignant are defined as smooth-muscle tumors of uncertain malignant potential (STUMPs).

Uterine sarcomas account for only 2–3% of uterine tumors, most of which are benign uterine leiomyomas^6^. Surgical treatments for uterine leiomyoma include myomectomy or total hysterectomy, depending on the patient's desire for preserving fertility. For uterine sarcomas, a total hysterectomy should be performed without fertility preservation because of the risk of tumor dissemination caused by dispersal in tumorectomy and morcellation^7,8^. However, unlike cancers of the uterine corpus and cervix, uterine sarcomas are difficult to biopsy, highlighting the importance of accurate preoperative diagnosis. As such, numerous studies have investigated strategies for improving the accuracy of diagnostic imaging for uterine sarcomas.

Computed tomography (CT), magnetic resonance imaging (MRI), and fluorodeoxyglucose-positron emission tomography-CT are reliable tools for diagnosing uterine sarcomas. Since there is no exposure to radiation, and the contrast resolution is high, MRI is considered the most reliable method; previous studies have elucidated several important MRI features of uterine sarcomas^9^. For example, the margin of uterine sarcomas is usually irregular, while that of uterine leiomyomas is well-defined. Furthermore, the T2-weighted imaging (T2WI) signal of uterine sarcomas is normally high when compared with the normal uterine myometrium, while that of uterine leiomyomas is low^10^. However, due the presence of overlapping imaging findings, differentiating between uterine sarcomas and leiomyomas on MRI can be challenging. Uterine leiomyomas with degeneration and cellular variants frequently mimic uterine sarcomas on MRI, and misdiagnosis of uterine sarcomas as benign leiomyomas is not uncommon^10–12^. Several studies have reported that these overlapping MRI features can lead to misdiagnosis of occult uterine sarcomas as benign tumors prior to surgery^13–15^. Conversely, some patients with uterine leiomyoma may undergo total hysterectomy due to overdiagnosis of uterine sarcoma^16^. Such reports demonstrate the critical impact of appropriate pre-treatment diagnosis in patients with uterine tumors.

Recent innovations in artificial intelligence (AI) and machine learning technology have advanced the medical field. Furthermore, significant improvements in computer hardware performance have led to the development of deep neural networks (DNNs)^17^. The accuracy of DNNs has exceeded that of conventional image processing methods at the ImageNet Large Scale Visual Recognition Challenge (ILSVRC)^18^, eventually surpassing the accuracy of human image recognition^19^. Despite this, large amounts of data are typically required to train DNN models, and their application in the diagnosis of rare diseases such as uterine sarcomas remains challenging.

Given their impressive capabilities, several research groups have aimed to develop machine learning methods for improving the accuracy of uterine tumor diagnosis using MRI. However, to our knowledge, none have utilized DNNs, which we believe can provide a diagnostic advantage because DNNs can learn with more parameters than conventional machine learning methods^11,20–25^. Therefore, in the current study, we aimed to investigate whether DNN models can be used to improve the accuracy of preoperative MRI-based diagnosis in patients with uterine sarcomas. In addition, we developed a method for improving the rate of accurate diagnosis for even a small number of cases. We also compared our DNN models with assessments performed by radiologists to determine their practicability. Notably, our study is the first to demonstrate the feasibility of DNN models for the diagnosis of uterine sarcomas using MR images. Our goal is to develop a universal model for the use of deep learning in the diagnostic imaging of rare tumors.

## Results

### Patients and MR images

Sixty-three cases of the two groups of uterine sarcomas and 200 cases of uterine leiomyomas were extracted from the three institutions. Supplementary Table 1 shows the histopathological types of uterine sarcomas included in this study. The frequency of LMS was consistent with the general frequency of 36 out of 63 cases (57%)^5^. Among the cases of uterine leiomyoma, 23 (11.5%) were diagnosed as uterine sarcomas preoperatively. The marginal and degeneration scores assigned by the six radiologists are shown in Supplementary Table 2. Degeneration alone, irregular margins alone, and both types of findings were noted in 66 (33%), 11 (5.5%), and nine (4.5%) cases, respectively. These results highlight the heterogenous characteristics of leiomyomas included in this study. Although 15 types of MRI sequences were adapted for learning and evaluation, none of the cases in our study had data for all 15 types of sequences. Supplementary Tables 3a and 3b show the numbers of patients and slices in each cross-validation group, respectively.

### Performance based on individual MRI sequences

**Figure 1:**
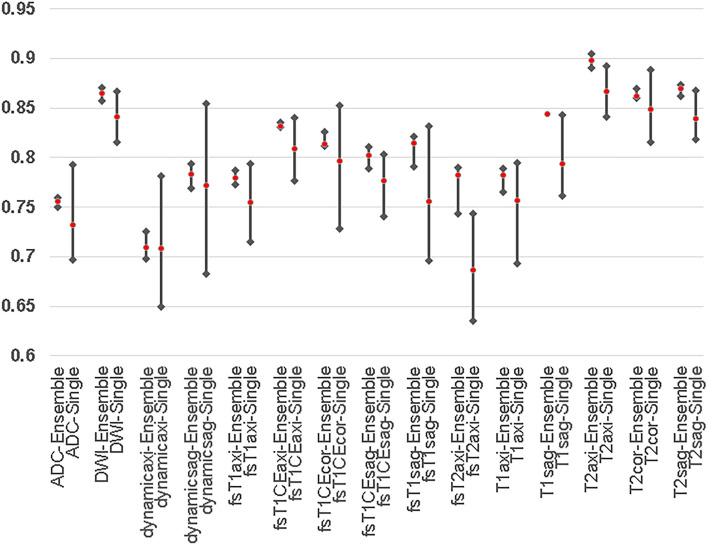
Results of DNN single-model and ensemble predictions of MRI sequences. The means and ranges of the SS-Avgs of the MRI sequences are shown. The top 3 MRI sequences of the ensemble predictions are T2axi (89.8%), T2sag (86.9%), and DWI (86.5%). The top 3 MRI sequences of the single-model predictions are T2axi (86.6%), T2cor (84.9%), and DWI (84.1%). The results of the ensemble predictions are better those from the single models for all MRI sequences.

Figure 1 shows the average of the sensitivity and specificity (SS-Avg) of each MRI sequence for the single-model predictions and the sets of ensemble predictions. For all sequences, the SS-Avgs show that the ensemble predictions performed better than the single-model predictions. Figure 1 also shows that the results from the ensemble predictions were more stable than those from single models. The top performers in terms of SS-Avg were T2axi (89.8%, SS-Avg), T2sag (86.9%, SS-Avg), and diffusion-weighted imaging (DWI) (86.5%, SS-Avg) for the ensemble predictions and T2axi (86.6%, SS-Avg), T2cor (84.9%, SS-Avg), and DWI (84.1%, SS-Avg) for the single-model predictions.

### Performance bases on combinations of MRI sequences

**Figure 2:**
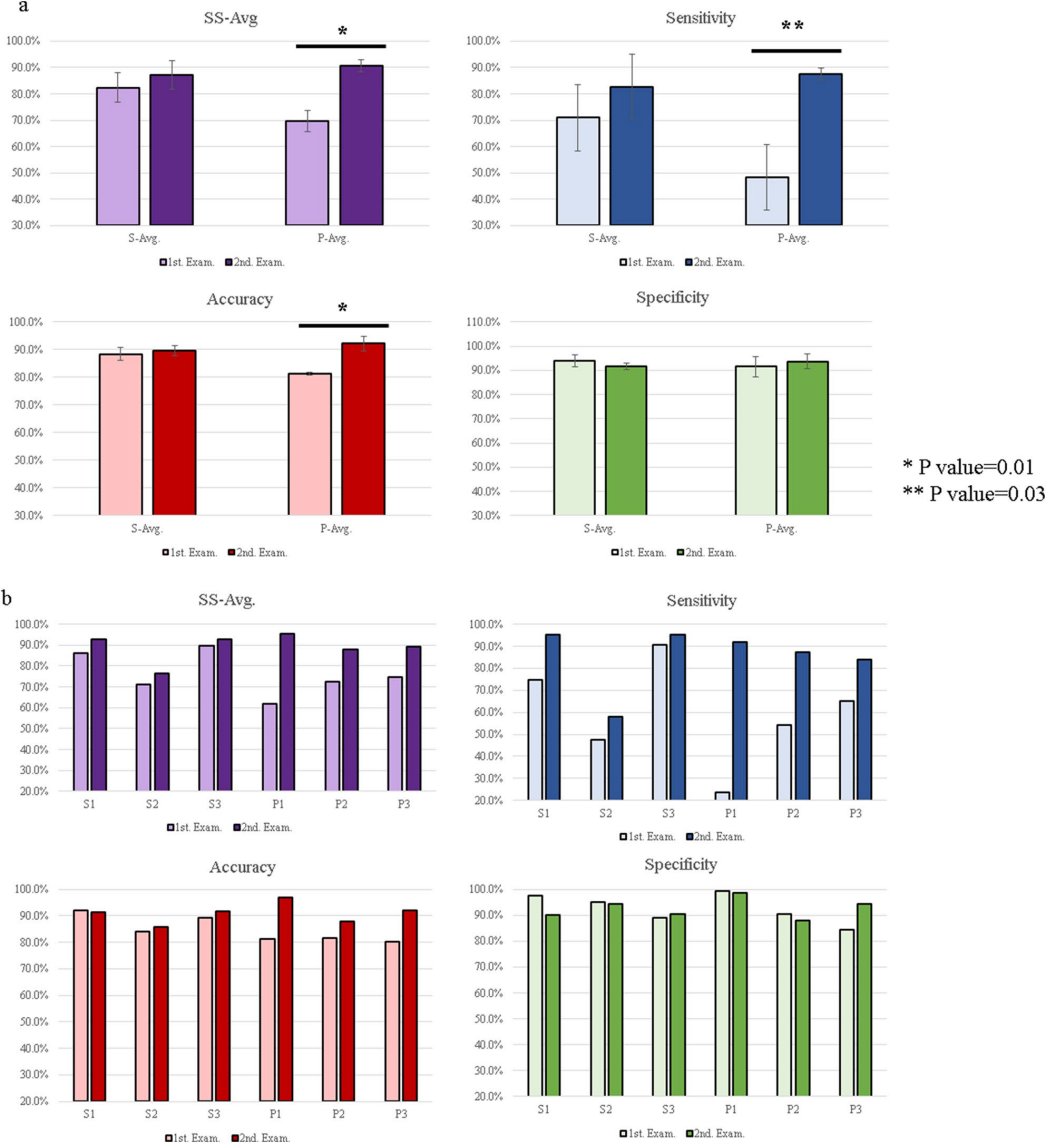

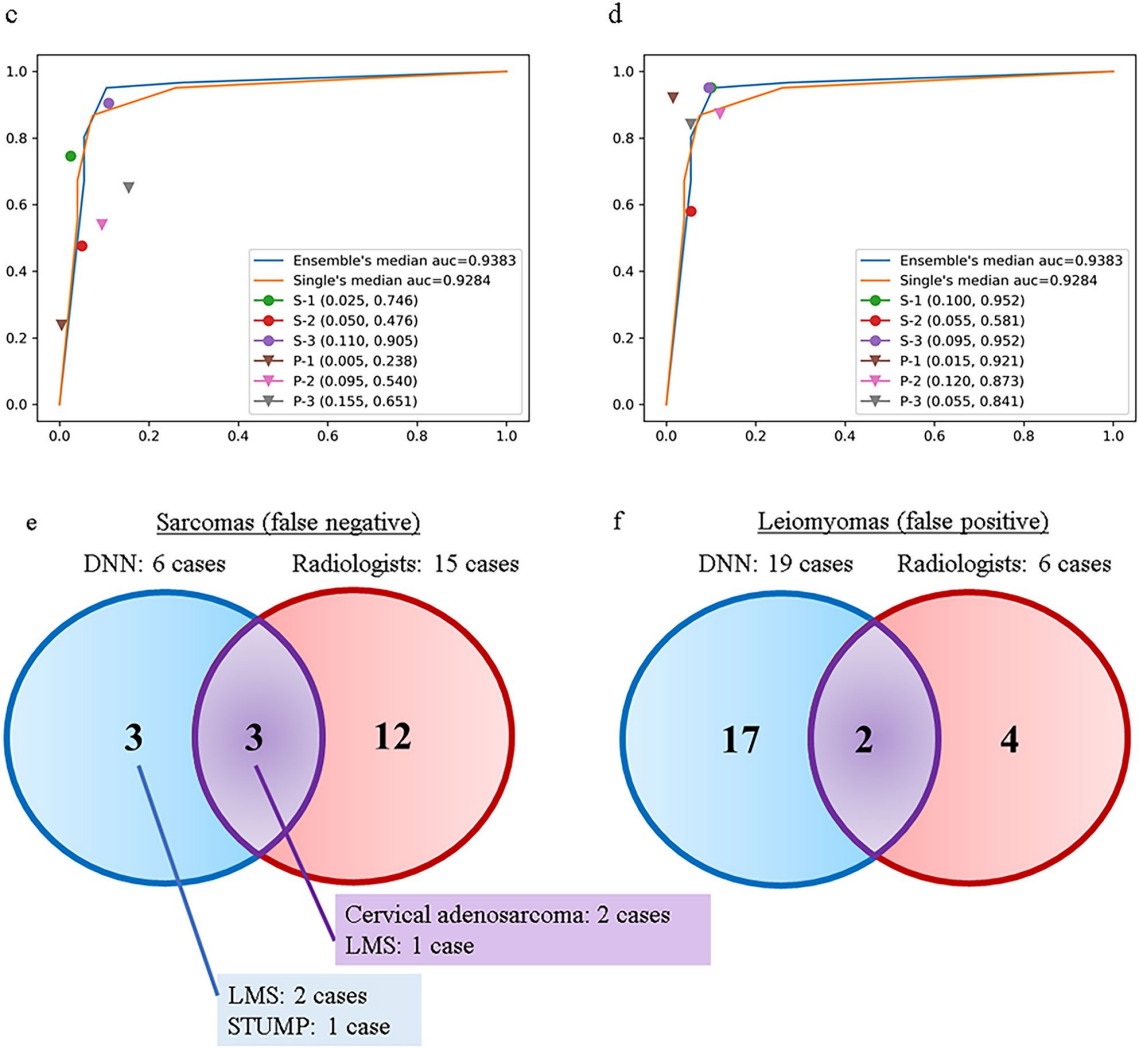
Comparison of diagnostic examinations. (**a**) Comparison between the first (no AI support) and second (AI-supported) diagnostic examinations for the data averages. For the radiological practitioners, all parameters show an increase between the first and second diagnostic examinations. The SS-Avg, accuracy, and sensitivity show significant increases (*p* < 0.05). Error bars indicate means ± SD. *p < 0.01, two-sided; **p < 0.05, two-sided. (**b**) Comparison between first (no AI support) and second (AI-supported) diagnostic examinations for the individual data. A comparison of the radiologist results between the first and the second diagnostic examinations show an increase in the SS-Avg and sensitivity for all radiologists. (**c**/**d**). ROC curves of individual results with of the DNN models. The figures show ROC curves of the individual results of the first (no AI support, **c**) and the second (AI-supported, **d**) diagnostic examinations. The ROC curves are based on the results of Combination Set 1 (shown in Table 1). The AI support improved the individual results. (**e**/**f**) Correlation diagrams of misdiagnosed images are shown for the uterine-sarcoma groups (**e**) and the uterine leiomyomas (**f**). The cases misdiagnosed by either the DNN models (blue circles), radiologists (red circles), or both (purple circles) are shown. The histopathological types of the false negatives from the DNN models included 1 case of STUMP, 2 cases of cervical adenosarcoma, and 3 cases of LMS.

The diagnostic results were improved by combining MRI sequences. Table 1 lists the combinations and grades of the top 10 MRI sequence combinations (out of 32,768 combination sets) in terms of SS-Avg. The average results for these 10 sets of combinations were adopted as the final results for our DNN models, which were provided to the radiologists in the second diagnostic examination (accuracy: 90.3%, SS-Avg: 90.8%, sensitivity: 89.8%, specificity: 91.7%, as shown in Table 1). The best MRI sequence combination for SS-Avg (combination set 1: T2axi, T2sag, and DWI) included the top three among the ensemble predictions. The diagnostic results for the ensemble predictions were also better than those for the single-model predictions, when combinations of MRI sequences were used. Supplementary Fig. 1 shows each ROC curve for the respective MRI sequences. The median area under the curve (AUC) for the ensemble predictions in combination set 1 was 0.9383, and the median AUC of individual models in combination set 1 was 0.9284 (Fig. 2c and d). The AUC values were also better for the ensemble predictions than for the single-model predictions. The average results for the combined MRI sequences (combination sets 1 to 10) indicated that the correct rate (sarcoma likelihood) was < 50% among the 6 cases of sarcoma (false negative) and > 50% among the 19 cases of leiomyoma (false positive). Among the 19 cases of leiomyoma, degeneration was noted in 12 cases (63.2%), and irregular margins were noted in one case (5.2%).

**Table 1:** Results of the DNN models for combinations of MRI sequences.

Combination set	SS-Avg (%)	Accuracy (%)	Sensitivity (%)	Specificity (%)	ADC	DWI	dynamicaxi	dynamicsag	fsT1axi	fsT1CEaxi	fsT1CEcor	fsT1CEsag	fsT1sag	fsT2axi	T1axi	T1sag	T2axi	T2cor	T2sag
Combination set1	91.3	89.9	88.7	94.0		●											●		●
Combination set2	91.3	90.5	89.8	92.9			●	●	●	●	●	●	●	●	●	●	●	●	●
Combination set3	91.1	91.5	91.9	90.3	●	●	●	●	●		●	●	●	●		●	●	●	●
Combination set4	91.0	89.3	87.8	94.2	●									●					●
Combination set5	90.8	90.7	90.7	90.9		●	●	●	●	●	●		●	●		●	●	●	
Combination set6	90.5	90.5	90.5	90.5		●	●	●	●	●	●		●	●		●	●	●	●
Combination set7	90.5	89.4	88.5	92.5	●	●	●	●	●	●	●	●		●		●	●	●	●
Combination set8	90.5	90.0	89.6	91.3	●		●	●	●	●	●	●	●	●		●	●	●	
Combination set9	90.4	89.7	89.0	91.8		●	●	●		●	●	●	●	●	●	●	●	●	●
Combination set10	90.3	91.1	91.9	88.8		●	●	●	●	●	●	●	●	●	●		●	●	●
**Average**	**90.8**	**90.3**	**89.8**	**91.7**															

The top 10 combinations of MRI sequences in terms of the SS-Avg (average of sensitivity and specificity) are shown. The average data for the top 10 combination sets were adopted as the results of the DNN models (accuracy: 90.3%; SS-Avg: 90.8%; sensitivity: 89.8%; specificity: 91.7%). Combination set 1 (T2axi, T2sag, and DWI) was the most accurate combination. DWI: diffusion-weighted imaging.

### Diagnostic interpretation by radiologists

To validate the quality of cases and images included in the current study, six radiologists performed diagnostic examinations of all 263 cases. The results of the first diagnostic examination without AI support are shown in Supplementary Table 4a. Table 2 lists a comparison of the results for radiological specialists, radiological practitioners, and the DNN models. In the first diagnostic examination, the results of radiological specialists were superior to those of radiological practitioners, and the DNN models performed significantly better in terms of SS-Avg and sensitivity, even when compared with radiological specialists (*p* < 0.05). After a 1-month interval, all six radiologists performed a second diagnostic examination in which they were provided with the interpretation of the DNN model (AI-supported examination). The individual results for the second diagnostic examination are shown in Supplementary Table 4b. The comparison between the first and second examinations is shown in Table 3 and Fig. 2a and b. SS-Avg, accuracy, and sensitivity increased significantly among radiological practitioners, while the increase among radiological specialists was not significant (*p* > 0.05). For all parameters, radiological practitioners performed better than radiological specialists in the AI-supported examination, although the difference was not significant (*p* > 0.05). In addition, increases in sensitivity and SS-Avg between the first and second examinations were observed for all radiologists. Indeed, after the AI-supported examination, many radiologists spent more time and were more careful when their interpretation did not match that indicated by the DNN models.

**Table 2:** Comparison between radiologists and DNN models in the first diagnostic examination (no AI support).

	S-Avg (%)	P-Avg (%)	DNN model (%)
SS-Avg	82.4	69.6	90.8
Accuracy	88.3	80.1	90.3
Sensitivity	71.0	47.6	89.8
Specificity	93.8	91.5	91.7

S-Avg: Average results for radiological specialists (board-certified radiologists).

P-Avg: Average results for radiological practitioners (no board-certified radiologists).

The results of DNN model were calculated using the average for the top 10 combinations of MRI sequences (Table 1).

The DNN results were equivalent to those of a radiological specialist, and the SS-Avg and sensitivity were significantly higher for the DNN models (*p* < 0.05 for both parameters).

**Table 3:** Comparison of parameters between the first and second (i.e., AI-supported) diagnostic examinations.

DNN model	SS-Avg (%)		Accuracy (%)		Sensitivity (%)		Specificity (%)	
	90.8		90.3		89.8		91.7	
	S-Avg	P-Avg	S-Avg	P-Avg	S-Avg	P-Avg	S-Avg	P-Avg
1st. Exam. (no AI support)	82.4	69.6	88.3	80.1	71.0	47.6	93.8	91.5
2nd. Exam. (with AI-supported)	87.3	90.8	89.6	92.3	83.1	87.8	91.7	93.7

S-Avg: Average results for radiological specialists.

P-Avg: Average results for radiological practitioners.

DNN model results (sarcoma likelihood and sequences for each patient) were provided to the radiologists for the second examination.

Remarkably, all parameters were superior among radiological practitioners than among radiological specialists, although the difference was not significant (*p* > 0.05).

Figure 2c and d also shows the comparison between the first and second examinations of each radiologist based on the ROC curve of the DNN models, again highlighting improvements in the AI-supported examination. Figure 2e and f shows the relationship between cases in which more than half of the 24 sets of DNN models and more than half of radiologists misdiagnosed the findings (false positive and false negative). The DNN models had a lower rate of false negatives and a higher rate of false positives than radiologists. An analysis of the relationship between the tumor diameter and the degeneration score among the 19 cases of false-positive uterine leiomyomas indicated that there was no bias in terms of tumor diameter for the DNN models, although 12 of 19 (63.2%) cases involved degeneration (Supplementary Fig. 2).

## Discussion

In this study, we investigated the usefulness of DNN models in differentiating between uterine sarcomas and uterine leiomyomas on MRI. Our analysis indicated that the DNN models achieved results comparable to those of the radiological specialists (DNN: 90.3% accuracy, 91.3% SS-Avg, 89.8% sensitivity, and 91.7% specificity; radiological specialist: 88.2% accuracy, 82.4% SS-Avg, 71.0% sensitivity, 93.8% specificity), although SS-Avg and sensitivity were significantly higher for the DNN models. In addition, radiological practitioners exhibited improvement in diagnostic skill to comparable levels with radiological specialists (accuracy 92.3% vs. 89.6%; SS-Avg 90.8% vs. 87.3%; sensitivity 87.8% vs. 82.8%; and specificity 93.7% vs. 91.7%) when provided with AI support. These findings highlight the usefulness of DNN models as diagnostic aids, suggesting that they can reduce the risk of misdiagnosis in patients with occult uterine sarcomas by improving sensitivity among both specialists and practitioners and fill the gaps between interpreters.

Several previous studies have indicated that AI support increases the diagnostic accuracy for uterine sarcomas^11,20–25^. Because uterine sarcomas are rare and previous reports included only a limited number of cases, a major strength of our study is that we used a relatively large number of cases and various of MRI sequences for model training, which can train DNN models with adequate number of images. Furthermore, to our knowledge, our study is the first to utilize DNN models for the diagnosis of uterine sarcoma by MRI. Conventional machine learning algorithms (i.e., non-DNN, Legacy-ML) include an extremely small number of parameters suitable for inputting learning/prediction when compared with DNN, and training conventional algorithms requires humans to determine the parameters to be learned and quantify them in advance. In contrast, DNN models such as MobileNet-V2 can include 50,176 (224 × 224) parameters, meaning that 224 × 224 pixels of the image can be input, allowing the model to learn and predict features that cannot be recognized by humans or are difficult to quantify. This is the greatest advantage of using DNN models; the current findings suggest the feasibility of DNN models for exceeding the accuracy of human interpretation in the future.

Since uterine sarcomas are rare, only a limited number of cases could be included in this study; this made the generation of DNN models challenging, given that large amounts of data/cases are required for model training. To overcome this, we utilized augmentation, ensemble predictions, a unique parameter “SS-Avg”, various MRI sequences with scoring system, and combinations of MRI sequences. The results of the ensemble predictions were superior to those of the single-model predictions in every sequence, and the ensemble predictions provided more-stable results than the single-model predictions. In addition, we evaluated our results using the SS-Avg value because it is influenced more by large values for sensitivity or specificity when they are imbalanced. In this study, we adapted “SS-Avg” to assess the well-balanced models capable of accurately diagnosing both uterine sarcomas and uterine leiomyomas. Although this is an uncommon method of evaluation, it is a useful strategy for investigating rare diseases because of the difficulty in balancing the number of cases between rare diseases and control cases. Moreover, since the imbalance of imaging condition among multi-institutions is critical for clinical adaptation, we have developed a score calculation for imbalanced combinations of MRI sequences provided by multi-institutions, which can be helpful in clinical practice.

Among the 15 sequences used in this study, the top results for the ensemble predictions were T2axi (89.8%, SS-Avg), T2sag (86.9%, SS-Avg), and DWI (86.5%, SS-Avg). Our findings also indicated that the top-performing combination of MRI sequences included these sequences (Table 1, combination set 1). T2axi (86.6%, SS-Avg) ranked first among the single-model predictions, while DWI (84.1%, SS-Avg) and T2sag (83.9%, SS-Avg) were ranked third and fourth, respectively. Interestingly, these combinations are clinically important, and a larger number of sequences in combination did not yield better results^26^. However, given the rarity of the disease, the number of sequences in each patient was imbalanced, and there were more images for these top three sequences in each patient than for other sequences (T2axi [258 cases, 98.0%], T2sag [259 cases, 98.4%], and DWI [254 cases, 96.5%] (Supplementary Table 3a and 3b), which should be considered when interpreting the findings**.**

When comparing misdiagnosed cases between the DNN models and radiologists, as well as false positives and false negatives, we observed that the DNN models provided substantial diagnostic assistance. As Fig. 2e and 4f shows, the DNN models had a lower rate of false negatives and a higher rate of false positives than the radiologists.

For the DNN models, the false negatives included two cases of cervical adenosarcoma and one case of STUMP. The tumor size of one cervical adenosarcoma was approximately 3 cm in diameter, and STUMPs are normally difficult to diagnose preoperatively, highlighting the need for future studies to develop more focused AI strategies^27^. Among the false-positive cases for the DNN models, 12 of 19 cases (63.2%) involved degeneration, while only 1 case (5.3%) had irregular margins. In addition, as shown in Supplementary Fig. 2, there was no bias associated with tumor diameter among false-positive cases for the DNN models. These findings suggest that DNN models identify sarcomas based on the degeneration inside the tumors rather than the tumor diameter. Visualizing how DNN models make decisions is critical for identifying areas for improvement.

The present study has some limitations, including the small number of patients given the rarity of the disease. Other limitations include an imbalance in the types of sequences and imaging conditions among the three institutions and the exclusion of patients with other abdominal tumors, such as ovarian tumors. Although the current study only involved learning and evaluation after cross-validation, we originally intended to prepare a validation set. This represents a limitation of DNN models when faced with a limited number of cases. Anatomically, uterine leiomyomas often coexist with other tumors such as ovarian endometrial cysts, and it remains necessary to distinguish leiomyomas from these other lesions. In addition, to maintain diagnostic accuracy, we limited our study to two types of output (uterine leiomyoma for negative or uterine sarcoma for positive). Further studies including a larger number of cases and more balanced imaging conditions are required to address these issues. Inclusion of additional clinical information (age, blood data, tumor markers, etc.) may also aid in the eventual diagnosis of individual histopathological types.

In summary, our analysis indicated that the DNN models developed in this study exhibited high-quality results for the diagnosis of uterine sarcomas using MRI images. We could develop a DNN model with an acceptable diagnostic rate for the rare uterine sarcoma tumors, and our method could be applied to the diagnoses of other rare tumors in the future. Specifically, AI support improved the sensitivity of interpretations made by radiologists, suggesting that DNN models can aid in reducing the risk of misdiagnosing occult uterine sarcomas. In the future, our MRI-based DNN system will be further developed and applied for uterine sarcoma diagnosis in clinical practice.

## Methods

### Patients

The current study included patients with uterine leiomyomas or sarcomas treated at three Japanese institutions (University of Tokyo Hospital, Tokyo Metropolitan Cancer and Infectious Diseases Center Komagome Hospital, and Showa General Hospital) from 2008 to 2020. All MR images were obtained during the study period and prior to tumor resection, and film-based MR imaging data were excluded.

**Figure 3:**
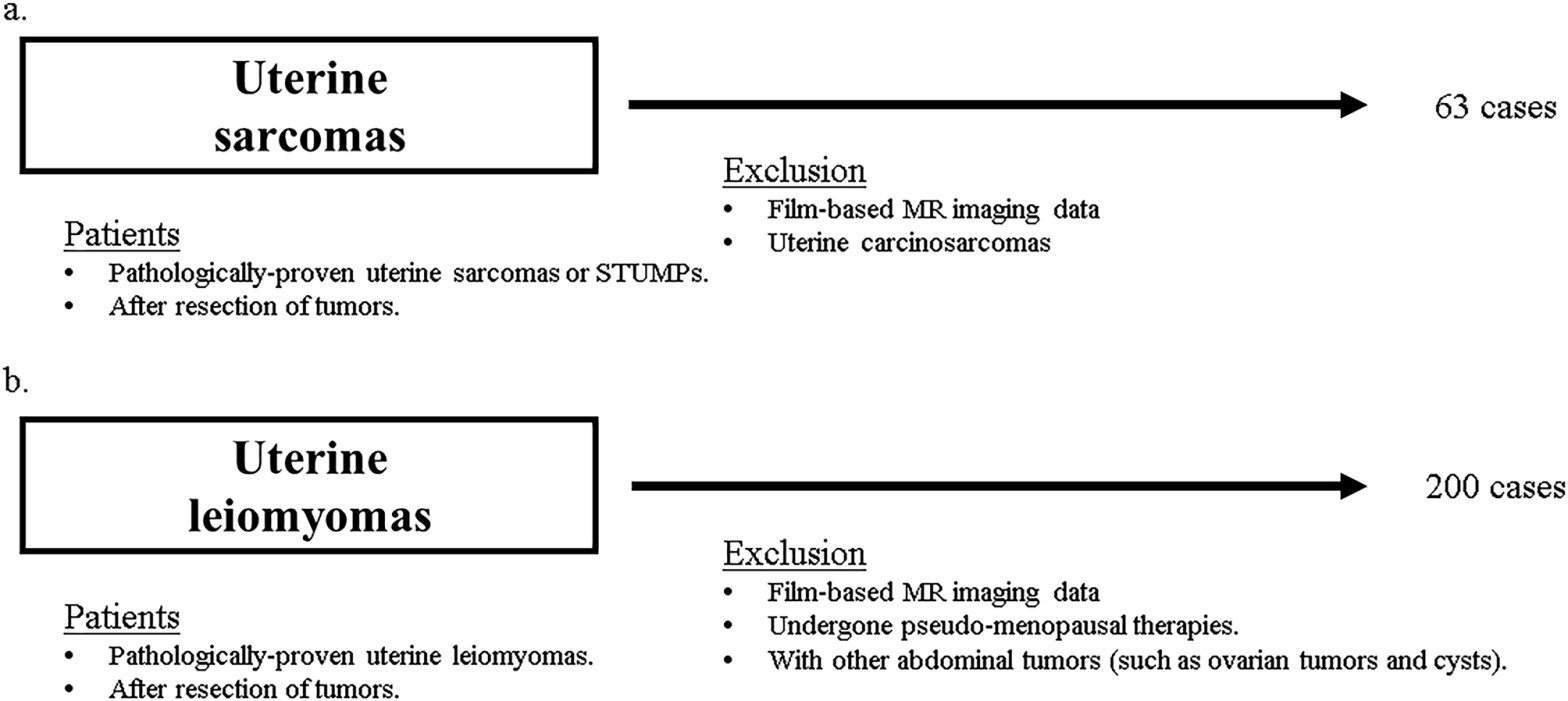
Study flow of patients. (**a**) Sixty-three cases of uterine sarcomas, including smooth muscle tumours of uncertain malignant potential (STUMPs), were included. The study excluded film-based MR images and carcinosarcomas. (**b**) Two-hundred cases of uterine leiomyomas sarcomas were included. The study excluded film-based MR images and patients who had undergone pseudo-menopausal therapies within 3 years or had other coexisting abdominal tumours.

To increase the number of training images, the uterine sarcoma set included multiple MR images obtained at different times for each patient. There were no restrictions on the preoperative period during which uterine sarcoma images were obtained. However, for the uterine leiomyoma image set, we extracted images that were taken within 1 year before resection. Patients who had undergone pseudo-menopause therapy within 3 years before resection, those with other co-existing tumors such as ovarian tumors, and those with ovarian cysts ≥ 3 cm were excluded. There were no restrictions on tumor number or diameter in either group. The detailed inclusion and exclusion criteria for each set of MR images are shown in Fig. 3.

All uterine tumors in this study were resected after MRI and pathologically diagnosed by well-trained pathologists. This study also included various histopathological types of uterine sarcomas groups including LMS, ESS, adenosarcoma, undifferentiated sarcoma, spindle-cell sarcoma, and smooth-muscle tumors of uncertain malignant potential (STUMPs). For the reasons indicated in the Introduction, carcinosarcomas were excluded.

### MR images

Fifteen types of MRI sequences were used, as shown in Supplementary Table 5. Other sequences were excluded. Six radiologists evaluated the tumor margins and degeneration of the uterine leiomyomas. Degeneration and margin irregularity were defined as cases in which at least four of six radiologists regarded the tumors as degeneration or margins as irregular, respectively. The imaging conditions for each MRI sequence are listed in Supplementary Table 6.

### Datasets

MRI slices including the uterine tumors were extracted for model learning and evaluation. First, we collected DICOM data from each institution and converted them into normalized JPEG data using the Horos software (https://horosproject.org/) with default settings. For cross-validation, we randomly divided patients into six groups and prepared six datasets, using five groups for learning and the remaining group for evaluation. Both groups were composed such that the number of slices was as uniform as possible, although the balance of MRI sequences was not considered.

### Type of neural network

In this study, we adopted the MobileNet-V2 network, which is a relatively compact network consisting of 88 layers with a fixed input image size of 224 × 224 and 3,538,984 learning parameters^28^. We adopted AMSGrad, a variant of Adam, as the optimizer, with the learning rate set to 0.0001. The structure of the network we adopted is shown in Supplementary Table 7.

### DNN learning

**Figure 4:**
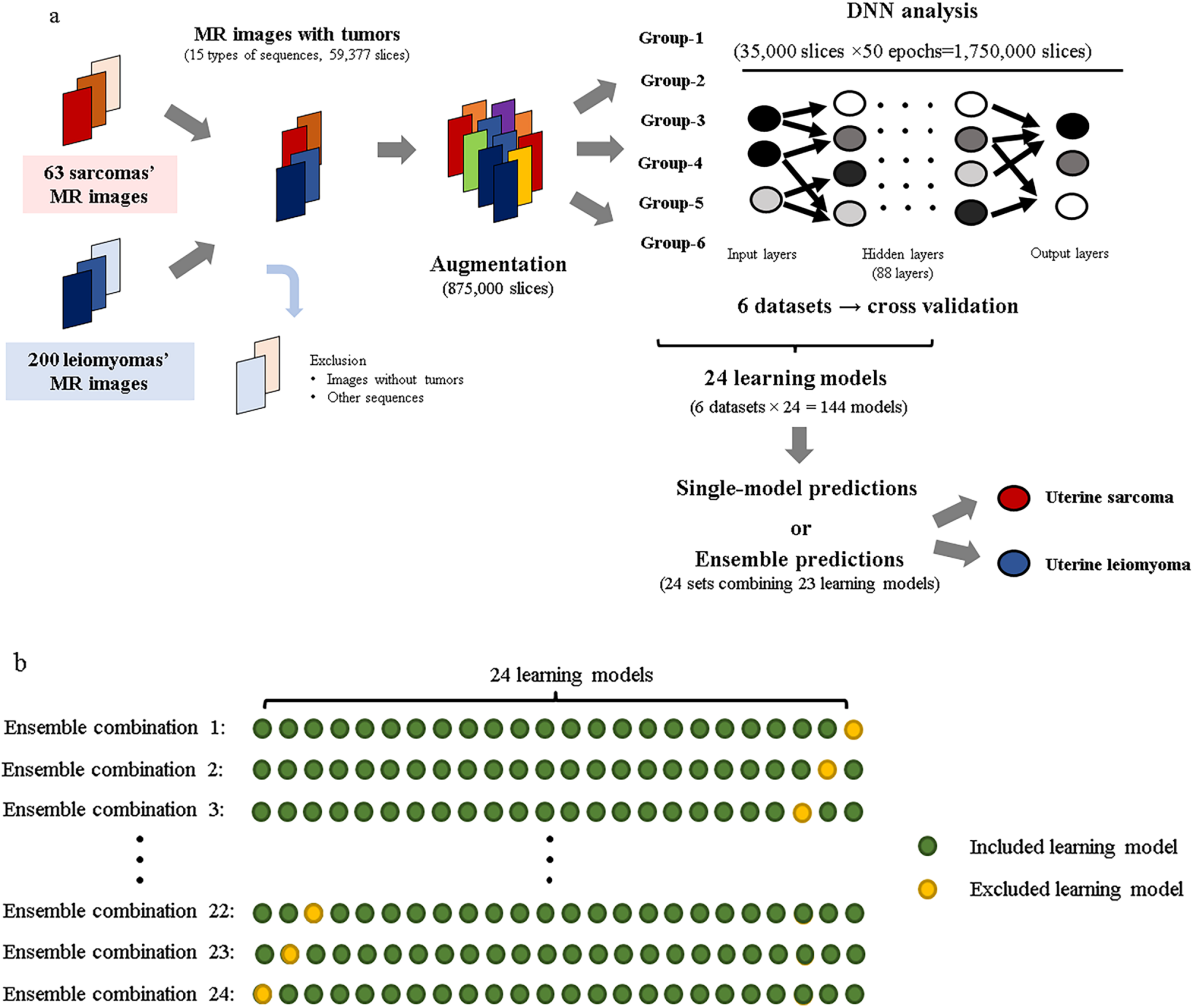
Study flow of DNN learning and evaluation. (**a**) MRI slices of uterine leiomyomas and sarcomas are augmented to 875,000 slices. In one epoch, 35,000 slices are selected randomly out of 875,000 slices and the model repeats learning 50 times. The ratio of the learning set to the evaluation set is 5:1, which is cross validated. The DNN models are evaluated as “a uterine sarcoma” or “a uterine leiomyoma” using either a single-model prediction or ensemble prediction. The augmentation method we adopted was a very general approach that included flips, rotations, zooms, and changes to the brightness. (**b**) For evaluation, 24 sets of ensemble predictions are performed along with single-model predictions. The predictions of the ensemble model combine the results of 23 models.

In this study, 437,500 MRI slices were augmented for both the uterine-sarcoma groups (sarcomas and STUMPs) and uterine leiomyomas. The augmentation was performed randomly without considering the balance between the number of MRI slices for each patient and each sequence. During learning, the DNN models learned using images cropped to a size of 224 × 224, keeping the tumor area of the image in the scope. In each epoch (training cycle), 35,000 slices were randomly selected from 875,000 slices, and 50 epochs were performed repeatedly to train one DNN model (35,000 slices × 50 = 1,750,000 slices). This 50-epoch training procedure was performed with six datasets, and six models were generated using one learning set (learning set: evaluation set = 5:1). Because DNN models exhibit differences in ability each time they are trained using a large amount of data generated via augmentation from a small number of patients, we created 24 training sets (M1 to M24) to verify the differences in the abilities of each model. As a result, 144 models were generated (6 datasets × 24 = 144 models). Figure 4a shows the flow of the model learning and evaluation process. The augmentation method we adopted was a very general approach that including flips, rotations, zooms, and changes to the brightness.

### DNN evaluation

We used square MR images that had been cropped and resized to 224 × 224. The six models obtained in each learning set were used as a single evaluation set, and predictions for the 24 evaluation sets were made based on single slices, single sequences, and combined sequences. In addition to single-model predictions, 24 sets (Ens1 to Ens24) of ensemble predictions combining 23 of the 24 models (Supplementary Table 8) were used to evaluate the results of the sequence-based and patient-based evaluations (Fig. 4b). Using these methods, we developed algorithms to classify images as uterine sarcomas or uterine leiomyomas, although we did not evaluate the histopathological types of uterine sarcomas.

The results obtained using the 24 sets of ensemble predictions were evaluated as percentages and defined as the possibility of uterine sarcoma, hereafter referred to as “sarcoma likelihood” (see Supplementary Table 9 for examples).

### Scoring

As this was a multi-institutional study, different MRI sequences were used to assess patients at each institution. Therefore, for each MRI sequence, scores of 1 and –1 were assigned to uterine sarcoma and uterine leiomyoma, respectively, and the total score was calculated to predict the result for each patient. A receiver operating characteristic (ROC) curve was used to identify the threshold value at which the average sensitivity (true positive [TP] rate) and specificity (true negative [TN] rate) were highest.

### Diagnostic examination by radiologists

Six radiologists, including three radiological specialists (board-certified radiologists, 14, 13, and 8 years of experience) and three radiological practitioners (no board-certified radiologists, 4, 4, and 2 years of experience), participated in diagnostic assessments to validate the quality of the MR images used in this study. In the first diagnostic examination (no AI support), the specialists interpreted all MR images learned and evaluated by the DNN models as either uterine sarcoma or uterine leiomyoma. After a 1-month break, radiologists were provided with the results of the DNN models (the sarcoma likelihood and sequence results for each patient) for a second, AI-supported examination, which was performed using the same procedure as the first examination. All diagnostic examinations were performed using anonymized and randomized data.

### Statistical analysis

Because the numbers of patients/slices were not the same for the uterine-sarcoma groups and uterine leiomyomas, conventional accuracy alone was not suitable for evaluating the usefulness of the DNN models, as it is more strongly influenced by large values for sensitivity or specificity. Therefore, we used the average of sensitivity and specificity (SS-Avg) as an additional parameter. Note that, when the numbers of patients/slices are the same for each group, SS-Avg and conventional accuracy should be equivalent. The final results of the DNN models were calculated by averaging the results for the top 10 MRI sequence combinations. In these analyses, the uterine-sarcoma groups were defined as positive, and the uterine leiomyomas were defined as negative. The methods used to calculate each parameter were as follows:

$$\begin{aligned} {\mathrm{Accuracy}} & = \, \left( {{\mathrm{TP }} + {\mathrm{ TN}}} \right) \, / \, \left( {{\mathrm{TP }} + {\mathrm{ TN }} + {\mathrm{ false positive }}\left[ {{\mathrm{FP}}} \right] \, + {\mathrm{ false negative }}\left[ {{\mathrm{FN}}} \right]} \right) \\ {\mathrm{SS}} - {\mathrm{Avg}} & = \, \left( {{\mathrm{TP }}/ \, \left( {{\mathrm{TP }} + {\mathrm{ FN}}} \right) \, + {\mathrm{ TN }}/ \, \left( {{\mathrm{TN }} + {\mathrm{ FP}}} \right)} \right) \, /{ 2} \\ {\mathrm{Sensitivity}} & = {\mathrm{ TP }}/ \, \left( {{\mathrm{TP}} + {\mathrm{FN}}} \right) \\ {\mathrm{Specificity}} & = {\mathrm{ TN }}/ \, \left( {{\mathrm{TN}} + {\mathrm{FP}}} \right) \\ \end{aligned}$$

In addition, the volume of uterine tumors was briefly calculated using the method described below. In cases with multiple tumors, the diameter of the largest tumor was measured.

$${\mathrm{Tumor\,volume}} = {3}/{4 } \times \, \pi \, \times {\mathrm{ a }} \times {\mathrm{ b }} \times {\mathrm{ c }}\left( {{\mathrm{a}} = {\mathrm{length}}/{2},{\mathrm{ b}} = {\mathrm{width}}/{2},{\mathrm{ c}} = {\mathrm{height}}/{2}} \right)$$

When comparing results between radiologists and the DNN models, significant differences in normally distributed data were defined using Welch's t-test for equal variance and Student’s *t*-test for unequal variance, both of which were two-sided. Statistical significance was set at *P* < 0.05.

### Ethics

This study was approved by the institutional review boards of each institution (Research ethics committee of the faculty of medicine of the University of Tokyo, Research ethics committee of Tokyo metropolitan cancer and infectious diseases center Komagome Hospital and Research ethics committee of Showa General Hospital),The institutional review board approval numbers are 2019127NI at the University of Tokyo Hospital, 2640 at the Tokyo Metropolitan Cancer and Infectious Diseases Center Komagome Hospital, and RED255 at the Showa General Hospital. In patient application forms, the need for informed consent was waived by these above institutional review boards. It was clearly stated that patients were allowed to opt out of the study at any time. Information on how they could opt out was provided on our website, and arrangements were made for patients to opt out. All methods were performed in accordance with the relevant guidelines and regulations.

## Supplementary Information

Supplementary Information 1.

Supplementary Information 2.

Supplementary Information 3.

Supplementary Information 4.

Supplementary Information 5.

Supplementary Information 6.

Supplementary Information 7.

Supplementary Information 8.

Supplementary Information 9.

Supplementary Information 10.

Supplementary Information 11.

## Data Availability

All data generated and analyzed during this study are included in this published article and its supplementary files.
