# Supplementary material for: Development of a deep learning method for improving diagnostic accuracy for uterine sarcoma cases
Source: Sci Rep. 2022 Nov 16;12:19612. doi: 10.1038/s41598-022-23064-5 (PMC9669038; doi:10.1038/s41598-022-23064-5)
Supplement: Supplementary file 10 — Supplementary Information 10. [file 41598_2022_23064_MOESM10_ESM.docx]

**Supplementary Information**

**Development of a deep learning method for improving diagnostic accuracy for rare tumors: uterine sarcoma cases**

Yusuke Toyohara ^1^, Kenbun Sone ^1^, Katsuhiko Noda ^2^, Kaname Yoshida ^2^, Ryo Kurokawa ^3^, Tomoya Tanishima ^3^, Shimpei Kato ^3^, Shohei Inui ^3^, Yudai Nakai ^3^, Masanori Ishida ^3^, Wataru Gonoi ^3^, Saki Tanimoto ^1^, Yu Takahashi ^1^, Futaba Inoue ^1^, Asako Kukita ^1^, Yoshiko Kawata ^4^, Ayumi Taguchi ^1^, Akiko Furusawa ^4^, Yuichiro Miyamoto ^1^, Takehiro Tsukazaki ^5^, Michihiro Tanikawa ^1^, Takayuki Iriyama ^1^, Mayuyo Mori-Uchino ^1^, Tetsushi Tsuruga ^1^, Katsutoshi Oda ^6^, Toshiharu Yasugi ^4^, Kimihiro Takechi ^5^, Osamu Abe ^3^, Yutaka Osuga ^1^

^1^ Department of Obstetrics and Gynecology, Graduate School of Medicine, The University of Tokyo, Tokyo, Japan

^2^ SIOS Technology, Inc., Tokyo, Japan

^3^ Department of Radiology, Graduate School of Medicine, The University of Tokyo, Tokyo, Japan

^4^ Department of Obstetrics and Gynecology, Tokyo Metropolitan Cancer and Infectious Diseases Center Komagome Hospital, Tokyo, Japan

^5^ Department of Obstetrics and Gynecology, Showa General Hospital, Tokyo, Japan.

^6^ Division of Integrative Genomics, Graduate School of Medicine, The University of Tokyo, Tokyo, Japan

***Corresponding author:**

Dr. Kenbun Sone

Assistant Professor, Department of Obstetrics and Gynecology

Faculty of Medicine, The University of Tokyo

7-3-1 Hongo Bunkyo-ku, Tokyo 113-8655, Japan

Phone: +81-3-3815-5411

Fax: +81-3-3816-2017

E-mail: [ksone5274@gmail.com](mailto:ksone5274@gmail.com)

**Supplementary Figures**

**Supplementary Figure 1.** ROC curves of MRI sequences

The ROC curves of the MRI sequences of the DNN models are shown. The ROC curves were calculated with the result of Combination Set 1 (T2axi, T2sag, and DWI). All AUCs of the ensemble predictions were better than the single-model predictions.

**Supplementary Figure 2.** Correlation between volume and degeneration score for misdiagnosed leiomyomas

Volumes of the tumors were calculated using the maximum length, width, and height of the tumors. The DNN models misdiagnosed 19 cases (false positives). Among the misdiagnosed uterine leiomyomas, various tumor sizes are included and the degeneration scores of 12/19 cases (63.2%) were positive (> 4 points).

**Supplementary Tables**

**Supplementary Table 1.** Histopathological types of the uterine-sarcoma groups

| Histopathology (Uterine sarcomas groups) | | No. of cases | |
| --- | --- | --- | --- |
| Leiomyosarcoma | | 36 | |
| Undifferentiated uterine sarcoma | | | 10 |
| Adenosarcoma | | | 6 |
| Endometrial stromal sarcoma | High-grade | | 4 |
|  | Low-grade | | 2 |
| Spindle-cell sarcoma | | | 2 |
| STUMP | | | 3 |
| Total | | | 63 |

STUMP: smooth muscle tumor of uncertain malignant potential

**Supplementary Table 2.** Degeneration and margin irregularity in uterine leiomyomas

|  |  | **Margin score**  (cases) | | **Total** |
| --- | --- | --- | --- | --- |
|  |  | **Negative**  (Regular) | **Positive**  (Irregular) |  |
| **Degeneration score**  (cases) | **Negative** | 132 (66%) | 2 (1%) | 134 (67%) |
|  | **Positive** | 57 (28.5%) | 9 (4.5%) | 66 (33%) |
| **Total** | | 189 (94.5%) | 11 (5.5%) | 200 (100%) |

Positive scores indicate cases in which more than 50% of the radiologists (4/6) identified irregular margins or degeneration.

**Supplementary Table 3a.** Numbers of cases of cross-validation groups (excel file)

**Supplementary Table 3b.** Numbers of slices of cross-validation groups (excel file)

Number of cases(3a) and slices(3b) of cross-validation groups. The balance of images was considered when grouping, however, the balance of sequences was not when grouping.

**Supplementary Table 4a.** Individual results for radiologists in the first diagnostic examination (no AI support)

| The first diagnostic examination (no AI support) | | | | | | |
| --- | --- | --- | --- | --- | --- | --- |
|  | **S-1** | **S-2** | **S-3** | **P-1** | **P-2** | **P-3** |
| SS-Avg | 86.1% | 71.3% | 89.8% | 61.7% | 72.3% | 74.8% |
| Accuracy | 92.0% | 84.0% | 89.4% | 81.3% | 81.7% | 80.2% |
| Sensitivity | 74.6% | 47.6% | 90.5% | 23.8% | 54.0% | 65.1% |
| Specificity | 97.5% | 95.0% | 89.0% | 99.5% | 90.5% | 84.5% |

S-1, S-2, S-3: Respective results for the three radiological specialists.

P-1, P-2, P-3: Respective results for the three radiological practitioners.

In the first diagnostic examination (no AI support), the radiologists interpreted the same MR images learned and evaluated using the DNN models as either uterine sarcoma or uterine leiomyoma.

**Supplementary Table 4b**. Individual results for radiologists in the second diagnostic examination (AI-supported)

| The second diagnostic examination (AI-supported) | | | | | | |
| --- | --- | --- | --- | --- | --- | --- |
|  | **S-1** | **S-2** | **S-3** | **P-1** | **P-2** | **P-3** |
| SS-Avg | 92.6% | 76.3% | 92.9% | 95.3% | 87.7% | 89.3% |
| Accuracy | 91.3% | 85.9% | 91.6% | 97.0% | 87.8% | 92.0% |
| Sensitivity | 95.2% | 58.1% | 95.2% | 92.1% | 87.3% | 84.1% |
| Specificity | 90.0% | 94.5% | 90.5% | 98.5% | 88.0% | 94.5% |

S-1, S-2, S-3: Respective results for the three radiological specialists.

P-1, P-2, P-3: Respective results for the three radiological practitioners.

The second diagnostic examination (AI-supported examination) was performed after a 1-month break, and the results of the DNN models (sarcoma likelihood and sequence results for each patient) are provided. Radiologists used the same procedures to evaluate images during the first and second examinations.

**Supplementary Table 5.** Types of MRI sequences and abbreviations

| Types of MRI sequences | Abbreviation |
| --- | --- |
| Axial T1-weighted image | T1axi |
| Sagittal T1-weighted image | T1sag |
| Fat suppressed axial T1-weighted image | fsT1axi |
| Fat suppressed sagittal T1-weighted image | fsT1sag |
| Axial T2-weighted image | T2axi |
| Sagittal T2-weighted image | T2sag |
| Coronal T2-weighted image | T2cor |
| Fat suppressed axial T2-weighted image | fsT2axi |
| Diffusion-weighted image | DWI |
| Apparent diffusion coefficient map image | ADC |
| Axial dynamic contrast-enhanced image | dynamicaxi |
| Sagittal dynamic contrast-enhanced image | dynamicsag |
| Axial fat suppressed contrast-enhanced T1-weighted image | fsT1CEaxi |
| Sagittal fat suppressed contrast-enhanced T1-weighted image | fsT1CEsag |
| Coronal fat suppressed contrast-enhanced T1-weighted image | fsT1CEcor |

On axial and sagittal dynamic contrast-enhanced T1WI, the images of the last phase (90–180 sec after contrast material injection) were collected. On DWI, images with high-b values (800–1500 s/mm^2^) were collected.

**Supplementary Table 6.** Imaging conditions for respective MRI sequences

(excel file)

MRI was performed at three different institutions with different imaging conditions.

**Supplementary Table 7.** The network structure of MobileNetV2.

| Layer (type) | Output Shape | Param# | Connected to |
| --- | --- | --- | --- |
| input_1 (InputLayer) | (None, 224, 224, 3) | 0 |  |
| Conv1_pad (ZeroPadding2D) | (None, 225, 225, 3) | 0 | input_1[0][0] |
| Conv1 (Conv2D) | (None, 112, 112, 32) | 864 | Conv1_pad[0][0] |
| bn_Conv1 (BatchNormalization) | (None, 112, 112, 32) | 128 | Conv1[0][0] |
| Conv1_relu (ReLU) | (None, 112, 112, 32) | 0 | bn_Conv1[0][0] |
| expanded_conv_depthwise (Depthw | (None, 112, 112, 32) | 288 | Conv1_relu[0][0] |
| expanded_conv_depthwise_BN (Bat | (None, 112, 112, 32) | 128 | expanded_conv_depthwise[0][0] |
| expanded_conv_depthwise_relu (R | (None, 112, 112, 32) | 0 | expanded_conv_depthwise_BN[0][0] |
| expanded_conv_project (Conv2D) | (None, 112, 112, 16) | 512 | expanded_conv_depthwise_relu[0][0 |
| expanded_conv_project_BN (Batch | (None, 112, 112, 16) | 64 | expanded_conv_project[0][0] |
| block_1_expand (Conv2D) | (None, 112, 112, 96) | 1536 | expanded_conv_project_BN[0][0] |
| block_1_expand_BN (BatchNormali | (None, 112, 112, 96) | 384 | block_1_expand[0][0] |
| block_1_expand_relu (ReLU) | (None, 112, 112, 96) | 0 | block_1_expand_BN[0][0] |
| block_1_pad (ZeroPadding2D) | (None, 113, 113, 96) | 0 | block_1_expand_relu[0][0] |
| block_1_depthwise (DepthwiseCon | (None, 56, 56, 96) | 864 | block_1_pad[0][0] |
| block_1_depthwise_BN (BatchNorm | (None, 56, 56, 96) | 384 | block_1_depthwise[0][0] |
| block_1_depthwise_relu (ReLU) | (None, 56, 56, 96) | 0 | block_1_depthwise_BN[0][0] |
| block_1_project (Conv2D) | (None, 56, 56, 24) | 2304 | block_1_depthwise_relu[0][0] |
| block_1_project_BN (BatchNormal | (None, 56, 56, 24) | 96 | block_1_project[0][0] |
| block_2_expand (Conv2D) | (None, 56, 56, 144) | 3456 | block_1_project_BN[0][0] |
| block_2_expand_BN (BatchNormali | (None, 56, 56, 144) | 576 | block_2_expand[0][0] |
| block_2_expand_relu (ReLU) | (None, 56, 56, 144) | 0 | block_2_expand_BN[0][0] |
| block_2_depthwise (DepthwiseCon | (None, 56, 56, 144) | 1296 | block_2_expand_relu[0][0] |
| block_2_depthwise_BN (BatchNorm | (None, 56, 56, 144) | 576 | block_2_depthwise[0][0] |
| block_2_depthwise_relu (ReLU) | (None, 56, 56, 144) | 0 | block_2_depthwise_BN[0][0] |
| block_2_project (Conv2D) | (None, 56, 56, 24) | 3456 | block_2_depthwise_relu[0][0] |
| block_2_project_BN (BatchNormal | (None, 56, 56, 24) | 96 | block_2_project[0][0] |
| block_2_add (Add) | (None, 56, 56, 24) | 0 | block_1_project_BN[0][0]  block_2_project_BN[0][0] |
| block_3_expand (Conv2D) | (None, 56, 56, 144) | 3456 | block_2_add[0][0] |
| block_3_expand_BN (BatchNormali | (None, 56, 56, 144) | 576 | block_3_expand[0][0] |
| block_3_expand_relu (ReLU) | (None, 56, 56, 144) | 0 | block_3_expand_BN[0][0] |
| block_3_pad (ZeroPadding2D) | (None, 57, 57, 144) | 0 | block_3_expand_relu[0][0] |
| block_3_depthwise (DepthwiseCon | (None, 28, 28, 144) | 1296 | block_3_pad[0][0] |
| block_3_depthwise_BN (BatchNorm | (None, 28, 28, 144) | 576 | block_3_depthwise[0][0] |
| block_3_depthwise_relu (ReLU) | (None, 28, 28, 144) | 0 | block_3_depthwise_BN[0][0] |
| block_3_project (Conv2D) | (None, 28, 28, 32) | 4608 | block_3_depthwise_relu[0][0] |
| block_3_project_BN (BatchNormal | (None, 28, 28, 32) | 128 | block_3_project[0][0] |
| block_4_expand (Conv2D) | (None, 28, 28, 192) | 6144 | block_3_project_BN[0][0] |
| block_4_expand_BN (BatchNormali | (None, 28, 28, 192) | 768 | block_4_expand[0][0] |
| block_4_expand_relu (ReLU) | (None, 28, 28, 192) | 0 | block_4_expand_BN[0][0] |
| block_4_depthwise (DepthwiseCon | (None, 28, 28, 192) | 1728 | block_4_expand_relu[0][0] |
| block_4_depthwise_BN (BatchNorm | (None, 28, 28, 192) | 768 | block_4_depthwise[0][0] |
| block_4_depthwise_relu (ReLU) | (None, 28, 28, 192) | 0 | block_4_depthwise_BN[0][0] |
| block_4_project (Conv2D) | (None, 28, 28, 32) | 6144 | block_4_depthwise_relu[0][0] |
| block_4_project_BN (BatchNormal | (None, 28, 28, 32) | 128 | block_4_project[0][0] |
| block_4_add (Add) | (None, 28, 28, 32) | 0 | block_3_project_BN[0][0]  block_4_project_BN[0][0] |
| block_5_expand (Conv2D) | (None, 28, 28, 192) | 6144 | block_4_add[0][0] |
| block_5_expand_BN (BatchNormali | (None, 28, 28, 192) | 768 | block_5_expand[0][0] |
| block_5_expand_relu (ReLU) | (None, 28, 28, 192) | 0 | block_5_expand_BN[0][0] |
| block_5_depthwise (DepthwiseCon | (None, 28, 28, 192) | 1728 | block_5_expand_relu[0][0] |
| block_5_depthwise_BN (BatchNorm | (None, 28, 28, 192) | 768 | block_5_depthwise[0][0] |
| block_5_depthwise_relu (ReLU) | (None, 28, 28, 192) | 0 | block_5_depthwise_BN[0][0] |
| block_5_project (Conv2D) | (None, 28, 28, 32) | 6144 | block_5_depthwise_relu[0][0] |
| block_5_project_BN (BatchNormal | (None, 28, 28, 32) | 128 | block_5_project[0][0] |
| block_5_add (Add) | (None, 28, 28, 32) | 0 | block_4_add[0][0]  block_5_project_BN[0][0] |
| block_6_expand (Conv2D) | (None, 28, 28, 192) | 6144 | block_5_add[0][0] |
| block_6_expand_BN (BatchNormali | (None, 28, 28, 192) | 768 | block_6_expand[0][0] |
| block_6_expand_relu (ReLU) | (None, 28, 28, 192) | 0 | block_6_expand_BN[0][0] |
| block_6_pad (ZeroPadding2D) | (None, 29, 29, 192) | 0 | block_6_expand_relu[0][0] |
| block_6_depthwise (DepthwiseCon | (None, 14, 14, 192) | 1728 | block_6_pad[0][0] |
| block_6_depthwise_BN (BatchNorm | (None, 14, 14, 192) | 768 | block_6_depthwise[0][0] |
| block_6_depthwise_relu (ReLU) | (None, 14, 14, 192) | 0 | block_6_depthwise_BN[0][0] |
| block_6_project (Conv2D) | (None, 14, 14, 64) | 12288 | block_6_depthwise_relu[0][0] |
| block_6_project_BN (BatchNormal | (None, 14, 14, 64) | 256 | block_6_project[0][0] |
| block_7_expand (Conv2D) | (None, 14, 14, 384) | 24576 | block_6_project_BN[0][0] |
| block_7_expand_BN (BatchNormali | (None, 14, 14, 384) | 1536 | block_7_expand[0][0] |
| block_7_expand_relu (ReLU) | (None, 14, 14, 384) | 0 | block_7_expand_BN[0][0] |
| block_7_depthwise (DepthwiseCon | (None, 14, 14, 384) | 3456 | block_7_expand_relu[0][0] |
| block_7_depthwise_BN (BatchNorm | (None, 14, 14, 384) | 1536 | block_7_depthwise[0][0] |
| block_7_depthwise_relu (ReLU) | (None, 14, 14, 384) | 0 | block_7_depthwise_BN[0][0] |
| block_7_project (Conv2D) | (None, 14, 14, 64) | 24576 | block_7_depthwise_relu[0][0] |
| block_7_project_BN (BatchNormal | (None, 14, 14, 64) | 256 | block_7_project[0][0] |
| block_7_add (Add) | (None, 14, 14, 64) | 0 | block_6_project_BN[0][0] |
|  |  |  | block_7_project_BN[0][0] |
| block_8_expand (Conv2D) | (None, 14, 14, 384) | 24576 | block_7_add[0][0] |
| block_8_expand_BN (BatchNormali | (None, 14, 14, 384) | 1536 | block_8_expand[0][0] |
| block_8_expand_relu (ReLU) | (None, 14, 14, 384) | 0 | block_8_expand_BN[0][0] |
| block_8_depthwise (DepthwiseCon | (None, 14, 14, 384) | 3456 | block_8_expand_relu[0][0] |
| block_8_depthwise_BN (BatchNorm | (None, 14, 14, 384) | 1536 | block_8_depthwise[0][0] |
| block_8_depthwise_relu (ReLU) | (None, 14, 14, 384) | 0 | block_8_depthwise_BN[0][0] |
| block_8_project (Conv2D) | (None, 14, 14, 64) | 24576 | block_8_depthwise_relu[0][0] |
| block_8_project_BN (BatchNormal | (None, 14, 14, 64) | 256 | block_8_project[0][0] |
| block_8_add (Add) | (None, 14, 14, 64) | 0 | block_7_add[0][0]  block_8_project_BN[0][0] |
| block_9_expand (Conv2D) | (None, 14, 14, 384) | 24576 | block_8_add[0][0] |
| block_9_expand_BN (BatchNormali | (None, 14, 14, 384) | 1536 | block_9_expand[0][0] |
| block_9_expand_relu (ReLU) | (None, 14, 14, 384) | 0 | block_9_expand_BN[0][0] |
| block_9_depthwise (DepthwiseCon | (None, 14, 14, 384) | 3456 | block_9_expand_relu[0][0] |
| block_9_depthwise_BN (BatchNorm | (None, 14, 14, 384) | 1536 | block_9_depthwise[0][0] |
| block_9_depthwise_relu (ReLU) | (None, 14, 14, 384) | 0 | block_9_depthwise_BN[0][0] |
| block_9_project (Conv2D) | (None, 14, 14, 64) | 24576 | block_9_depthwise_relu[0][0] |
| block_9_project_BN (BatchNormal | (None, 14, 14, 64) | 256 | block_9_project[0][0] |
| block_9_add (Add) | (None, 14, 14, 64) | 0 | block_8_add[0][0]  block_9_project_BN[0][0] |
| block_10_expand (Conv2D) | (None, 14, 14, 384) | 24576 | block_9_add[0][0] |
| block_10_expand_BN (BatchNormal | (None, 14, 14, 384) | 1536 | block_10_expand[0][0] |
| block_10_expand_relu (ReLU) | (None, 14, 14, 384) | 0 | block_10_expand_BN[0][0] |
| block_10_depthwise (DepthwiseCo | (None, 14, 14, 384) | 3456 | block_10_expand_relu[0][0] |
| block_10_depthwise_BN (BatchNor | (None, 14, 14, 384) | 1536 | block_10_depthwise[0][0] |
| block_10_depthwise_relu (ReLU) | (None, 14, 14, 384) | 0 | block_10_depthwise_BN[0][0] |
| block_10_project (Conv2D) | (None, 14, 14, 96) | 36864 | block_10_depthwise_relu[0][0] |
| block_10_project_BN (BatchNorma | (None, 14, 14, 96) | 384 | block_10_project[0][0] |
| block_11_expand (Conv2D) | (None, 14, 14, 576) | 55296 | block_10_project_BN[0][0] |
| block_11_expand_BN (BatchNormal | (None, 14, 14, 576) | 2304 | block_11_expand[0][0] |
| block_11_expand_relu (ReLU) | (None, 14, 14, 576) | 0 | block_11_expand_BN[0][0] |
| block_11_depthwise (DepthwiseCo | (None, 14, 14, 576) | 5184 | block_11_expand_relu[0][0] |
| block_11_depthwise_BN (BatchNor | (None, 14, 14, 576) | 2304 | block_11_depthwise[0][0] |
| block_11_depthwise_relu (ReLU) | (None, 14, 14, 576) | 0 | block_11_depthwise_BN[0][0] |
| block_11_project (Conv2D) | (None, 14, 14, 96) | 55296 | block_11_depthwise_relu[0][0] |
| block_11_project_BN (BatchNorma | (None, 14, 14, 96) | 384 | block_11_project[0][0] |
| block_11_add (Add) | (None, 14, 14, 96) | 0 | block_10_project_BN[0][0]  block_11_project_BN[0][0] |
| block_12_expand (Conv2D) | (None, 14, 14, 576) | 55296 | block_11_add[0][0] |
| block_12_expand_BN (BatchNormal | (None, 14, 14, 576) | 2304 | block_12_expand[0][0] |
| block_12_expand_relu (ReLU) | (None, 14, 14, 576) | 0 | block_12_expand_BN[0][0] |
| block_12_depthwise (DepthwiseCo | (None, 14, 14, 576) | 5184 | block_12_expand_relu[0][0] |
| block_12_depthwise_BN (BatchNor | (None, 14, 14, 576) | 2304 | block_12_depthwise[0][0] |
| block_12_depthwise_relu (ReLU) | (None, 14, 14, 576) | 0 | block_12_depthwise_BN[0][0] |
| block_12_project (Conv2D) | (None, 14, 14, 96) | 55296 | block_12_depthwise_relu[0][0] |
| block_12_project_BN (BatchNorma | (None, 14, 14, 96) | 384 | block_12_project[0][0] |
| block_12_add (Add) | (None, 14, 14, 96) | 0 | block_11_add[0][0]  block_12_project_BN[0][0] |
| block_13_expand (Conv2D) | (None, 14, 14, 576) | 55296 | block_12_add[0][0] |
| block_13_expand_BN (BatchNormal | (None, 14, 14, 576) | 2304 | block_13_expand[0][0] |
| block_13_expand_relu (ReLU) | (None, 14, 14, 576) | 0 | block_13_expand_BN[0][0] |
| block_13_pad (ZeroPadding2D) | (None, 15, 15, 576) | 0 | block_13_expand_relu[0][0] |
| block_13_depthwise (DepthwiseCo | (None, 7, 7, 576) | 5184 | block_13_pad[0][0] |
| block_13_depthwise_BN (BatchNor | (None, 7, 7, 576) | 2304 | block_13_depthwise[0][0] |
| block_13_depthwise_relu (ReLU) | (None, 7, 7, 576) | 0 | block_13_depthwise_BN[0][0] |
| block_13_project (Conv2D) | (None, 7, 7, 160) | 92160 | block_13_depthwise_relu[0][0] |
| block_13_project_BN (BatchNorma | (None, 7, 7, 160) | 640 | block_13_project[0][0] |
| block_14_expand (Conv2D) | (None, 7, 7, 960) | 153600 | block_13_project_BN[0][0] |
| block_14_expand_BN (BatchNormal | (None, 7, 7, 960) | 3840 | block_14_expand[0][0] |
| block_14_expand_relu (ReLU) | (None, 7, 7, 960) | 0 | block_14_expand_BN[0][0] |
| block_14_depthwise (DepthwiseCo | (None, 7, 7, 960) | 8640 | block_14_expand_relu[0][0] |
| block_14_depthwise_BN (BatchNor | (None, 7, 7, 960) | 3840 | block_14_depthwise[0][0] |
| block_14_depthwise_relu (ReLU) | (None, 7, 7, 960) | 0 | block_14_depthwise_BN[0][0] |
| block_14_project (Conv2D) | (None, 7, 7, 160) | 153600 | block_14_depthwise_relu[0][0] |
| block_14_project_BN (BatchNorma | (None, 7, 7, 160) | 640 | block_14_project[0][0] |
| block_14_add (Add) | (None, 7, 7, 160) | 0 | block_13_project_BN[0][0]  block_14_project_BN[0][0] |
| block_15_expand (Conv2D) | (None, 7, 7, 960) | 153600 | block_14_add[0][0] |
| block_15_expand_BN (BatchNormal | (None, 7, 7, 960) | 3840 | block_15_expand[0][0] |
| block_15_expand_relu (ReLU) | (None, 7, 7, 960) | 0 | block_15_expand_BN[0][0] |
| block_15_depthwise (DepthwiseCo | (None, 7, 7, 960) | 8640 | block_15_expand_relu[0][0] |
| block_15_depthwise_BN (BatchNor | (None, 7, 7, 960) | 3840 | block_15_depthwise[0][0] |
| block_15_depthwise_relu (ReLU) | (None, 7, 7, 960) | 0 | block_15_depthwise_BN[0][0] |
| block_15_project (Conv2D) | (None, 7, 7, 160) | 153600 | block_15_depthwise_relu[0][0] |
| block_15_project_BN (BatchNorma | (None, 7, 7, 160) | 640 | block_15_project[0][0] |
| block_15_add (Add) | (None, 7, 7, 160) | 0 | block_14_add[0][0]  block_15_project_BN[0][0] |
| block_16_expand (Conv2D) | (None, 7, 7, 960) | 153600 | block_15_add[0][0] |
| block_16_expand_BN (BatchNormal | (None, 7, 7, 960) | 3840 | block_16_expand[0][0] |
| block_16_expand_relu (ReLU) | (None, 7, 7, 960) | 0 | block_16_expand_BN[0][0] |
| block_16_depthwise (DepthwiseCo | (None, 7, 7, 960) | 8640 | block_16_expand_relu[0][0] |
| block_16_depthwise_BN (BatchNor | (None, 7, 7, 960) | 3840 | block_16_depthwise[0][0] |
| block_16_depthwise_relu (ReLU) | (None, 7, 7, 960) | 0 | block_16_depthwise_BN[0][0] |
| block_16_project (Conv2D) | (None, 7, 7, 320) | 307200 | block_16_depthwise_relu[0][0] |
| block_16_project_BN (BatchNorma | (None, 7, 7, 320) | 1280 | block_16_project[0][0] |
| Conv_1 (Conv2D) | (None, 7, 7, 1280) | 409600 | block_16_project_BN[0][0] |
| Conv_1_bn (BatchNormalization) | (None, 7, 7, 1280) | 5120 | Conv_1[0][0] |
| out_relu (ReLU) | (None, 7, 7, 1280) | 0 | Conv_1_bn[0][0] |
| global_average_pooling2d | (Globa (None, 1280) | 0 | out_relu[0][0] |
| dense (Dense) | (None, 1024) | 1311744 | global_average_pooling2d[0][0] |
| dense_1 (Dense) | (None, 2) | 2050 | dense[0][0] |

This table shows the network structure of MobileNetV2, which consists of 88 layers with a fixed input image size of 224 × 224 and 3,538,984 learning parameters.

**Supplementary Table 8.** The ensemble combination of 24 training sets.

| Ensemble Name | Model-set used for Ensemble |
| --- | --- |
| Ens1 | M1, M2, M3, M4, M5, M6, M7, M8, M9, M10, M11, M12, M13, M14, M15, M16, M17, M18, M19, M20, M21, M22, M23 |
| Ens2 | M2, M3, M4, M5, M6, M7, M8, M9, M10, M11, M12, M13, M14, M15, M16, M17, M18, M19, M20, M21, M22, M23, M24 |
| Ens3 | M3, M4, M5, M6, M7, M8, M9, M10, M11, M12, M13, M14, M15, M16, M17, M18, M19, M20, M21, M22, M23, M24, M1, |
| Ens4 | M4, M5, M6, M7, M8, M9, M10, M11, M12, M13, M14, M15, M16, M17, M18, M19, M20, M21, M22, M23, M24, M1, M2 |
| Ens5 | M5, M6, M7, M8, M9, M10, M11, M12, M13, M14, M15, M16, M17, M18, M19, M20, M21, M22, M23, M24, M1, M2, M3 |
| Ens6 | M6, M7, M8, M9, M10, M11, M12, M13, M14, M15, M16, M17, M18, M19, M20, M21, M22, M23, M24, M1, M2, M3, M4 |
| Ens7 | M7, M8, M9, M10, M11, M12, M13, M14, M15, M16, M17, M18, M19, M20, M21, M22, M23, M24, M1, M2, M3, M4, M5 |
| Ens8 | M8, M9, M10, M11, M12, M13, M14, M15, M16, M17, M18, M19, M20, M21, M22, M23, M24, M1, M2, M3, M4, M5, M6 |
| Ens9 | M9, M10, M11, M12, M13, M14, M15, M16, M17, M18, M19, M20, M21, M22, M23, M24, M1, M2, M3, M4, M5, M6, M7 |
| Ens10 | M10, M11, M12, M13, M14, M15, M16, M17, M18, M19, M20, M21, M22, M23, M24, M1, M2, M3, M4, M5, M6, M7, M8 |
| Ens11 | M11, M12, M13, M14, M15, M16, M17, M18, M19, M20, M21, M22, M23, M24, M1, M2, M3, M4, M5, M6, M7, M8, M9 |
| Ens12 | M12, M13, M14, M15, M16, M17, M18, M19, M20, M21, M22, M23, M24, M1, M2, M3, M4, M5, M6, M7, M8, M9, M10 |
| Ens13 | M13, M14, M15, M16, M17, M18, M19, M20, M21, M22, M23, M24, M1, M2, M3, M4, M5, M6, M7, M8, M9, M10, M11 |
| Ens14 | M14, M15, M16, M17, M18, M19, M20, M21, M22, M23, M24, M1, M2, M3, M4, M5, M6, M7, M8, M9, M10, M11, M12 |
| Ens15 | M15, M16, M17, M18, M19, M20, M21, M22, M23, M24, M1, M2, M3, M4, M5, M6, M7, M8, M9, M10, M11, M12, M13 |
| Ens16 | M16, M17, M18, M19, M20, M21, M22, M23, M24, M1, M2, M3, M4, M5, M6, M7, M8, M9, M10, M11, M12, M13, M14 |
| Ens17 | M17, M18, M19, M20, M21, M22, M23, M24, M1, M2, M3, M4, M5, M6, M7, M8, M9, M10, M11, M12, M13, M14, M15 |
| Ens18 | M18, M19, M20, M21, M22, M23, M24, M1, M2, M3, M4, M5, M6, M7, M8, M9, M10, M11, M12, M13, M14, M15, M16 |
| Ens19 | M19, M20, M21, M22, M23, M24, M1, M2, M3, M4, M5, M6, M7, M8, M9, M10, M11, M12, M13, M14, M15, M16, M17 |
| Ens20 | M20, M21, M22, M23, M24, M1, M2, M3, M4, M5, M6, M7, M8, M9, M10, M11, M12, M13, M14, M15, M16, M17, M18 |
| Ens21 | M21, M22, M23, M24, M1, M2, M3, M4, M5, M6, M7, M8, M9, M10, M11, M12, M13, M14, M15, M16, M17, M18, M19 |
| Ens22 | M22, M23, M24, M1, M2, M3, M4, M5, M6, M7, M8, M9, M10, M11, M12, M13, M14, M15, M16, M17, M18, M19, M20 |
| Ens23 | M23, M24, M1, M2, M3, M4, M5, M6, M7, M8, M9, M10, M11, M12, M13, M14, M15, M16, M17, M18, M19, M20, M21 |
| Ens24 | M24, M1, M2, M3, M4, M5, M6, M7, M8, M9, M10, M11, M12, M13, M14, M15, M16, M17, M18, M19, M20, M21, M22 |

This table shows the ensemble combination of 24 training sets. Twenty-four sets (Ens1 to Ens24) of ensemble predictions combining 23 of the 24 models were used to evaluate the results of the sequence-based and patient-based evaluations.

**Supplementary Table 9.** Examples of “sarcoma likelihood”

(excel file)

Results are provided for ensemble predictions. The “sarcoma likelihood” was determined based on the percentage of sets (sets 1 to 24) yielding a diagnosis of uterine sarcoma. These data were provided to radiologists for the second, AI-supported, diagnostic examination. S: uterine sarcoma; L: uterine leiomyoma
